# Assessment of the Popularity and Perceived Effectiveness of Smartphone Tools That Track and Limit Smartphone Use: Survey Study and Machine Learning Analysis

**DOI:** 10.2196/38963

**Published:** 2022-10-20

**Authors:** Elias Aboujaoude, Germano Vera Cruz, Lucien Rochat, Robert Courtois, Farah Ben Brahim, Riaz Khan, Yasser Khazaal

**Affiliations:** 1 Stanford University School of Medicine Department of Psychiatry and Behavioral Sciences Stanford, CA United States; 2 Department of Psychology University of Picardie Jules Verne Amiens France; 3 Addiction Division, Department of Psychiatry University Hospitals of Geneva Geneva Switzerland; 4 Department of Psychology Tours University Tours France; 5 Addiction Psychiatry Foederatio Medicorum Helveticorum Geneva Switzerland; 6 Lausanne University Lausanne Switzerland

**Keywords:** smartphone addiction, internet addiction, internet gaming disorder, smartphone tools, telepsychiatry, machine learning, telemedicine, social media, digital mental health interventions, mobile phone

## Abstract

**Background:**

Problematic smartphone use, like problematic internet use, is a condition for which treatment is being sought on the web. In the absence of established treatments, smartphone-provided tools that monitor or control smartphone use have become increasingly popular, and their dissemination has largely occurred without oversight from the mental health field.

**Objective:**

We aimed to assess the popularity and perceived effectiveness of smartphone tools that track and limit smartphone use. We also aimed to explore how a set of variables related to mental health, smartphone use, and smartphone *addiction* may influence the use of these tools.

**Methods:**

First, we conducted a web-based survey in a representative sample of 1989 US-based adults using the crowdsourcing platform Prolific. Second, we used machine learning and other statistical tools to identify latent user classes; the association between latent class membership and demographic variables; and any predictors of latent class membership from covariates such as daily average smartphone use, social problems from smartphone use, smartphone *addiction,* and other psychiatric conditions.

**Results:**

Smartphone tools that monitor and control smartphone use were popular among participants, including parents targeting their children; for example, over two-thirds of the participants used sleep-related tools. Among those who tried a tool, the highest rate of perceived effectiveness was 33.1% (58/175). Participants who experienced problematic smartphone use were more likely to be younger and more likely to be female. Finally, 3 latent user classes were uncovered: nonusers, effective users, and ineffective users. Android operating system users were more likely to be nonusers, whereas younger adults and females were more likely to be effective users. The presence of psychiatric symptoms did not discourage smartphone tool use.

**Conclusions:**

If proven effective, tools that monitor and control smartphone use are likely to be broadly embraced. Our results portend well for the acceptability of mobile interventions in the treatment of smartphone-related psychopathologies and, potentially, non–smartphone-related psychopathologies. Better tools, targeted marketing, and inclusive design, as well as formal efficacy trials, are required to realize their potential.

## Introduction

### Background

The recognition of psychological downsides to internet-related technologies is >2 decades old. A large body of epidemiological, phenomenological, and biological research has accumulated during that period, leading to the inclusion of *Gaming Disorder* in the International Classification of Diseases, 11th revision [1], and of *Internet Gaming Disorder* in the appendix to the Diagnostic and Statistical Manual of Mental Disorders (DSM), 5th Edition [2]. Although the field may have become better at identifying internet-related psychopathology and, in some cases, attaching an accepted diagnostic label to it, agreed upon treatment guidelines remain elusive.

### Conventional Treatments

Psychopharmacological interventions have been inspired by conditions to which problematic internet use has been compared, including obsessive-compulsive disorder, substance use disorders, behavioral addictions, and attention deficit hyperactivity disorder [3]. However, the relatively limited exploration of serotonin reuptake inhibitors [4], mu receptor antagonists [5], and stimulants [6] has not yielded solid evidence to support their broad use. Psychotherapeutic interventions—individual, group, and residential—have received more research attention [7] and possess a larger evidence base, especially in favor of cognitive behavioral therapy. However, methodological differences, sample nonrepresentativeness, and other research study limitations preclude strong conclusions and recommendations for wider adoption.

### Using Technology Against Itself

In this relative treatment vacuum, and in parallel with growing social and cultural recognition of the risks to personal well-being of runaway smartphone reliance, a new help modality has emerged and rapidly asserted itself among technology users and developers, with little direct contribution to its growth and design from clinicians and mental health experts. Described as “using technology against itself” [8], it involves 2 basic offerings: functionalities built into the smartphone that can be activated at will to monitor and limit use; and apps (dubbed “apps to wean us off apps”) that can be downloaded from a third party and used for the same purpose. Like the *old* psychopharmacological and psychotherapeutic interventions explored for problematic internet use, the goal is to track and curtail excessive or problematic smartphone use through tools that, if proven successful, might possess some unique advantages over traditional interventions, including scalability, cost-effectiveness, diminished stigma, convenience, and lack of side effects [8]. Aligned with mobile therapy, these interventions may also benefit from the big acceptability gains that the telepsychiatry field has enjoyed among patients and providers during the COVID-19 pandemic, further propelling their growth in the years to come.

This study assessed the use of built-in smartphone tools meant to monitor and target problematic or excessive smartphone use among US-based users. Examples of such tools include screen time (tracks and quantifies use), grayscale (makes apps and alerts less noticeable), disabling notifications (reduces distractions), audio messaging (limits typing and reduces confusion), night shift (reduces exposure to sleep-disrupting blue light), tools that move apps from the home screen to reduce distractibility, and tools that delete apps. Examining the popularity of such tools, users’ experiences with them, and any associations with demographic, mental health, and psychosocial factors can shed light on the promise and limitations of this growing field and help start a much-needed assessment by the mental health community of its place within the broader treatment landscape.

To do so, this study aimed to assess the prevalence of problematic smartphone use in a representative sample of US-based users, as well as the use of tools to monitor and control smartphone use (TMCSU). The study also aimed to explore how a set of variables related to mental health, smartphone use, and smartphone *addiction* may influence the use of TMCSU.

Toward that goal, we tried to answer 6 research questions (RQs), presented in Textbox 1.

**Research questions.**

**Research questions regarding a set of variables affecting use of tools to monitor and control smartphone use (TMCSU):**

1. Research question (RQ) 1: In a representative sample of users in the United States, what is the prevalence of problematic smartphone use, as measured by variables such as daily average use, daily average use for nonessential activities, social problems because of smartphone use, and smartphone *addiction*?
2. RQ2: In a representative sample of users in the United States, how common is the use of TMCSU and what is the perceived effectiveness of these tools? The tools are designed to, for instance, measure daily smartphone use duration, manage notifications, block problematic apps, improve sleep time and quality, and monitor and control smartphone use in underage children.
3. RQ3: In a representative sample of users in the United States, can we identify the underlying latent classes of smartphone users using TMCSU?
4. RQ4: If the underlying latent classes of smartphone users are identified, what are the associations between the participants’ sociodemographic characteristics (age and sex) and the uncovered latent classes?
5. RQ5: Among variables such as participants’ smartphone operating system, daily average smartphone use, social problems owing to smartphone use, smartphone *addiction*, and diagnosed mental health disorders, what are the most important features (covariates) predicting smartphone users’ latent class membership?
6. RQ6: What are the associations between the covariates (smartphone operating system, daily average smartphone use, social problems owing to smartphone use, smartphone *addiction*, mental health disorders, etc) and the latent classes associated with the use of TMCSU?

As this was designed as an exploratory study, no hypotheses were associated with these RQs.

To our knowledge, and despite the popularity of smartphone tools intended to curb smartphone use, this is the first psychological assessment of this digital mental health intervention. As such, our findings can help guide a field that is growing rapidly but mostly outside of meaningful scrutiny by the mental health scientific community.

## Methods

### Participants

A representative sample of the adult population in the United States was recruited. Overall, 1989 individuals participated in the survey and answered a web-based questionnaire. To be included, participants had to be adults (≥18 years), belong to a Prolific representative sample of the adult population in the United States, and provide digital informed consent for study participation. The only exclusion criterion was reporting no smartphone use.

The 2 sociodemographic characteristics collected were age and sex. Age varied between 19 and 76 years (mean 45, SD 16 years) and was distributed as follows: 19 to 35 years (691/1989, 34.74%); 36 to 60 years (802/1989, 40.32%); and 61 to 76 years (496/1989, 24.93%). The sex distribution was as following: male (965/1989, 48.51%); female (1006/1989, 50.57%); and nonbinary (18/1989, 0.9%).

### Recruitment and Sampling

The recruitment of study participants was conducted anonymously using the web-based crowdsourcing platform Prolific [9]. Prolific has been described as possessing some advantages over other similar platforms, including that it is exclusively dedicated to research studies, and its participants are more ethnically and geographically diverse and naive to experimental research tasks [10]. As such, it can allow the recruitment of a US-based sample of adults aged ≥18 years with sex, age, and ethnicity characteristics that reflect the US Census Bureau data. According to the Prolific platform, only people who have an account on Prolific are notified of studies that they are eligible for based on the demographic information they provide. When the study was posted on Prolific, an invitation was sent by Prolific to a random subset of all eligible individuals. To be eligible for a US representative sample, Prolific participants must be residents of the United States and be fluent in English. The sample in this study is approximately the maximum deliverable representative sample size by Prolific. Of the whole sample, 2% were excluded from the analyses because they did not use a smartphone. Participants received approximately US $1.3 for completing the questionnaire. The recruitment was launched and completed in March 2021.

### Data Collection Material

Data were collected using a web-based questionnaire. The questionnaire included 80 questions divided into 5 parts. The first part included 48 questions assessing sociodemographic characteristics (eg, age and sex); a screening question about smartphone use; items on smartphone operating system (OS); items on smartphone use behavior during the previous 12 months (eg, daily use duration, use behavior, daily average use duration on nonessential activities, and social problems owing to smartphone use); items on the use of smartphone TMCSU behavior during the previous 12 months (whether participants used them, and, if yes, how often [rarely, sometimes, or frequently] and with what perceived effectiveness [very effective, somewhat effective, neither effective nor ineffective, somewhat ineffective, or very ineffective]); and items on lifetime mental health and alcohol use disorder diagnoses (yes or no).

The second and third parts of the questionnaire assessed mental health and smartphone addiction, respectively. The latter was assessed using the smartphone application-based addiction scale [11], which comprises 6 items and a 6-point response scale ranging from *strongly disagree* to *strongly agree*. Mental health–related symptoms were assessed using the DSM, 5th Edition self-rated level 1 cross-cutting symptom measure [12] (24 items and a 5-point response scale ranging from *not at all* to *nearly every day*), which assesses symptoms over the 2 weeks preceding survey completion across 14 domains: depression, anxiety, anger, mania, personality functioning, sleep problems, somatic symptoms, suicidal ideation, psychosis, sleep problems, memory, repetitive thoughts and behaviors, and dissociation and substance use. In this study, these dimensions were labeled *DSM Depression*, *DSM Anxiety*, and so on.

The fourth part included a single item on how smartphone use may have changed during the COVID-19 pandemic (7-point response scale ranging from *decrease* to *increase*). Finally, the fifth part of the questionnaire included a single question about daily smartphone screen time. The raw data and collection materials were accessible through a coauthor’s research database [13].

### Data Analysis

To answer RQ1, we built a cross tab with the problematic smartphone use variables against sociodemographic characteristic variables, and we conducted a Pearson chi-square analysis on the resulting contingency table.

To answer RQ2, descriptive statistical analyses were conducted on the data regarding participants’ use of TMCSU.

To answer RQ3, we conducted latent class analysis (LCA) using the *poLCA* R package. LCA is a way to uncover hidden groupings within data. From the participants’ item responses, LCA algorithms divided participants into subgroups based on unobservable constructs (latent variables). The resulting subgroups were called latent classes. This technique is analogous to factor analysis where the model determines the latent variables from the manifest (measured) variables. Unlike cluster analysis techniques, which are based on mathematical distances (eg, Euclidean distances) or mathematical density, LCA is based on participants’ probability of giving different designed modalities of response (items-response probability) and the probability of membership in the modeled latent classes. Therefore, LCA is considered more advantageous than cluster analysis for model selection and interpretation [14,15]. In total, we ran 9 LCA models, with the first model nclass=2, second model nclass=3, and so on. In each model, the other poLCA function parameter settings were as follows: nrep=10, na.rm=F, graphs=T, and maxiter=100,000. After ensuring that the 9 models built were well identified (through maximum likelihood estimation), we proceeded with the comparison and model selection. On the basis of the Akaike information criterion, the Bayesian information criterion, entropy metrics (respectively 30224.17, 30858.43, and 0.83), and interpretability, we selected the model with 3 latent classes.

To answer RQ4, we computed a cross tab with age against sex against latent class, and then calculated Pearson chi-square of independence.

To answer RQ5, we built a machine learning model using the random forest (RF) classification algorithm [16]. The RF method uses a random subset of predictors and participants and, through recursive partitioning, tests the strength of each available predictor variable individually. This involves building a decision tree from the strongest available predictors and testing the tree’s overall predictive power on the *out-of-bag* sample (a subset of data that were not used to build the tree). The RF algorithm performs this repeatedly, separately bootstrapping thousands of decision trees and then averaging them out. RF classification models reveal, among other outputs, the importance of each predictor variable (predictors which made the largest contributions to the model) based on a measure called mean decrease accuracy (MDA). The MDA plot expresses how much accuracy the model loses by excluding each variable. The more the accuracy suffers, the more important the variable is for successful classification. Thus, the variables can be presented in ascending or descending order of importance. RF is nonparametric and, in essence, is able to capture nonlinear relationships [16]. To select the best model, we constructed 4 classification models with different fitting parameters. Each model was built using the *randomForest* package for R. In machine learning, the original data set is split into at least 2 sets: one to train the model (train-set; usually 70%-80% of the sample), the other to estimate the performance of the model when used to make predictions (test-set; 20%-30% of the sample). In this study, the data set was split as follows: train-set=70% and test-set=30% of the sample. The selected model had the following tuning parameters: *ntree*=500, which means that each RF model was built from 500 classification trees; and *mtry*=8, which means that the number of predictors available for splitting at each tree node was set to 8. The performance metrics of the selected model on the test-set data were as follows: accuracy score=0.73 (95% CI 0.66-0.78); no information rate=69; *P* (accuracy>no information rate)=0.038; κ=0.74.

The choice of using machine learning algorithms instead of traditional methods stems from the fact that these algorithms have hyperparameters that can be used to build different models with improved prediction capabilities to test the models’ respective performance using a subset of the main data set (named test-set) and to choose the models that best fit the data according to specific metrics [16]. Although the data set used in this study is relatively small for machine learning apps, the algorithm we used in our analysis (RF) is considered to be among the best for prediction analysis and for generating statistics of the most important predictor variables in ranking order [16]. Importantly, the RF algorithm has specific parameters that can be used to control the data set size and the imbalanced number of participants in the studied classes [16]. Furthermore, regarding prediction analysis, RF has been found to outperform traditional methods even when using relatively small data sets [16]. However, machine learning classification and regression algorithms are designed for prediction purposes and do not offer inference statistics; thus, we resorted to traditional methods such as logistic regression to obtain inference information (variable association probability metrics).

To answer RQ6, we built a multinomial logistic regression model using the SPSS software (version 28.0; IBM Corp). According to the likelihood ratio chi-square test, the full model showed a significant improvement in fit over the null model (*χ*^2^_36_=552.1, *P*<.001). Pearson chi-square test indicated that the model fit the data well (*χ*^2^_3880_=4031.4, *P*=.05), and the deviance chi-square indicated good fit (*χ*^2^_3880_=3490.9, *P*=.99)—indeed, in the latter 2 cases, nonsignificant test results were indicators that the model fit the data well (Field, 2018; Petrucci, 2009) [17,18].

### Ethics Approval

Participants provided digital informed consent for their survey contribution. Participation was voluntary and was restricted to those aged ≥18 years. All the data were collected anonymously. In accordance with the Swiss Human Research Act (Chapter 1, Section 1, Article 2 Scope: 2c) [19], no ethics assessment was applied for as anonymously collected or anonymized health-related data do not fall under the research act's scope.

## Results

### Basic Descriptive Statistics on the Participants Smartphone Use

The 2 sociodemographic characteristics collected were age and sex. Age varied between 19 and 76 years (mean 45, SD 16 years) and was distributed as follows: 19 to 35 (691/1989, 34.74%); 36 to 60 (802/1989, 40.32%); and 61 to 76 years (496/1989, 24.93%). The sex distribution was as follows: male (965/1989, 48.51%); female (1006/1989, 50.57%); and nonbinary (18/1989, 0.9%). The participants’ smartphone OS distribution was as follows: Android, 55.15% (1097/1989); iOS, 43.94% (874/1989); other, 0.3% (6/1989); and do not know, 0.6% (12/1989). Participants’ daily average smartphone use in the 12 months preceding the study was as follows: 0.27 to 17 hours (mean 3.33, SD 2.27 h; median=3 h). Furthermore, of 1989 participants’ daily average nonessential smartphone use in the 12 months preceding the study was as follows: <1 hour, 14.03% (279/1989); 1 to 3 hours, 39.22% (780/1989); 3 to 5 hours, 26.49% (527/1989); 5 to 7 hours, 9.45% (188/1989); 7 to 9 hours, 5.68% (113/1989); and >9 hours, 5.13% (102/1989). Finally, of the 1989 participants’ experience of social problems owing to smartphone use in the 12 months preceding the study was as follows: definitely no, 1109 (55.76%); probably no, 557 (28%); probably yes, 262 (13.17%); and definitely yes, 61 (3.07%) respectively.

### The Prevalence of Smartphone Problematic Use (RQ1)

Table 1 shows a cross tab of participant responses to the problematic smartphone use variables × the sociodemographic variables.

As shown in Table 1, 21.87% (435/1989) of the participants reported a high (4-17 h) daily average smartphone use; females and younger adults (19-35 years) were significantly more likely to be part of this group than males, adults (36-60 years), and older adults (61-76 years). A total of 46.76% (930/1989) of participants reported a high (>3 hours) daily average duration using the smartphone for nonessential activities; females and younger adults were more likely to be part of this group than males, adults, and older adults. Moreover, 16.24% (323/1989) of the participants reported having experienced social problems owing to problematic smartphone use; younger adults were more likely to be part of this group than adults and older adults. Finally, 13.42% (267/1989) of the participants reported experiencing smartphone *addiction* (scored >4 points, 1-6–point scale, in ≥4 of the 6 items in the smartphone application-based addiction scale); females and younger adults were more likely to be part of this group than males, adults, and older adults.

**Table 1:** Problematic smartphone use behavior in the US adult population by age category and sex (N=1989).

Demographics			DASU^a^, n (%)							DADUSNA^b^, n (%)					ESPDSU^c^, n (%)					SA^d^, n (%)		
			Low		Intermediate		High		Low			High		No			Yes		No			Yes
Total			554 (27.9)		1000 (50.3)		435 (21.9)		1059 (53.2)			930 (46.8)		1666 (83.8)			323 (16)		1722 (86.6)			267 (13.4)
**Age in years**																						
	19-35	106 (19.1)^e^		339 (33.9)^e^		246 (56.6)^e^		229 (21.6)^e^			462 (49.7)^e^		480 (28.8)^e^			211 (65.3)^e^		542 (31.4)^e^			149 (55.8)^e^	
	36-60	249 (44.9)^e,f^		409 (40.9)^e^		144 (33.1)^e^		456 (43.1)^e^			346 (37.2)^e^		707 (42.4)^f^			95 (29.4)^e^		705 (40.9)^e^			97 (36.3)^e^	
	61-76	199 (35.9)^e,g^		252 (25.2)^e^		45 (10.3)^e^		374 (35.3)^e^			122 (13.1)^e^		479 (28.8)^g^			17 (5.3)^e^		475 (27.6)^e^			21 (7.9)^e^	
**Sex**																						
	Male	320 (57.8)^e^		455 (45.5)^e^		190 (43.7)^e^		552 (52.1)^e^			413 (44.4)^e^		806 (48.4)^e^			159 (49.2)^e^		843 (49)^e^			122 (45.7)^e^	
	Female	231 (41.7)^e^		534 (53.4)^e^		241 (55.4)^e^		501 (47.3)^e^			505 (54.3)^e^		847 (50.8)^f^			159 (49.2)^f^		864 (50.1)^f^			142 (53.2)^e^	
	Nonbinary	3 (0.5)^e^		11 (1.1)^e^		4 (0.9)^e^		6 (0.6)^e^			12 (1.3)^e^		13 (0.8)^g^			5 (1.5)^g^		15 (0.9)^g^			3 (1.1)^f^	

^a^DASU: daily average smartphone use duration (low=0-2 h; intermediate≥2-4 h; >4-17 h).

^b^DADUSNA: daily average duration of using smartphones for nonessential activities (low=0-3 h; high≥3 h).

^c^ESPDSU: experienced social problems owing to smartphone use (in previous 12 months).

^d^SA: experiencing smartphone *addiction*; scored >4 points (on a 1-6–point scale) for ≥4 of the 6 items in the smartphone application-based addiction scale.

^e^Figures with the same exponent in each column were significantly different (*P*<.05). The figures with different exponents were not significantly different. For example, regarding age, 19% is significantly different from 45% and from 36%; 45% and 36% are not significantly different.

^f^Regarding age, 45% is significantly different from 19%.

^g^Regarding age, 36% is significantly different from 19%.

### The Popularity of TMCSU (RQ2)

As shown in Table 2, the 3 most commonly used tools were those designed to reduce notifications (973/1989, 48.92%), reduce smartphone screen time (913/1989, 45.9%), and improve sleep time and quality (702/1989, 35.29%). Of the tools that the participants tried, the most frequently used were designed to help sleep (484/1989, 24.33%) and reduce notifications (436/1989, 21.92%), whereas the ones considered the most effective were those that removed apps from the home screen (81/291, 27.8% found it effective), deleted apps (126/574, 21.9%), and helped sleep (147/702, 20.9%). Among the parents of underage children (483/1989, 24.28%), 36.2% (175/483) targeted their children with TMCSU, with 57.1% (100/175) using them frequently and 33.1% (58/175) finding them effective.

**Table 2:** Participants’ use of tools to monitor and control smartphone use, by tool (N=1989).

Tool category	Participants (N)	Participants using the tool, n (%)	Participants using the tool frequently^a^, n (%)	Participants that considered the tool effective^a^, n (%)
Tools to limit daily smartphone use duration	1989	263 (13.2)	29 (11)	39 (14.8)
Tools to reduce screen time	1989	913 (45.9)	96 (10.5)	155 (17)
Tools to calculate screen time	1989	676 (34)	96 (14.2)	115 (17)
Tools to block apps	1989	179 (9)	45 (25.1)	23 (12.8)
Tools make the smartphone less distracting	1989	115 (5.8)	39 (33.9)	17 (14.8)
Tools to improve sleep time and quality	1989	702 (35.2)	484 (68.9)	147 (20.9)
Tools to reduce notifications	1989	973 (48.9)	436 (44.8)	185 (19)
Tool to remove apps from smartphone home screen	1989	291 (14.6)	78 (26.8)	81 (27.8)
Tool to delete apps from smartphone	1989	574 (28.9)	113 (19.7)	126 (22)
Tool to control children’s smartphone use	483^b^	175 (36.2)	100 (57.1)	58 (33.1)

^a^Among the number of participants using the tool.

^b^Number of participants with (<18 years) children.

### Composition of the TMCSU Users’ Latent Classes (RQ3 and RQ4)

Table 3 shows the composition of the TMCSU latent classes by age and sex. The first latent class (691/1989, 34.74%) was labeled *nonsmartphone-use control* (NSC) because members of this group had a low or nonexistent probability of using any of the proposed TMCSU. Males, adults, and older adults were significantly more likely to be part of this group compared with females and younger adults. The second latent class (950/1989, 47.76%) was labeled as *ineffective–smartphone-use control* (ISC) because members of this group had a moderate probability of using any of the proposed TMCSU and tended to consider that the use of these tools was ineffective. Females, younger adults, and adults were more likely to be part of this group than males and older adults.

The third latent class (348/1989, 17.49%) was labeled *effective–smartphone-use control* (ESC) because members of this group had a moderate to high probability of using most of the proposed TMCSU and tended to consider that the use of these tools was effective. Females and younger adults were more likely to be part of this group than males, adults, and older adults.

**Table 3:** Composition of the tools to monitor and control smartphone use user latent classes by age category and sex (N=1989).

Demographics		Latent classes, n (%)				Total participants (N=1989), n (%)
		NSC^a^ (n=691)	ISC^b^ (n=950)	ESC^c^ (n=348)		
**Age (years)**						
	19-35	136 (19.7)^d^	350 (36.8)^d^	205 (59)^d^	691 (34.7)	
	36-60	291 (42.1)^d^	399 (42)^d^	112 (32.1)^d^	802 (40.3)	
	61-76	264 (38.2)^d,e^	201 (21.1)^d^	31 (9)^d^	496 (25)	
**Sex**						
	Male	356 (51.5)^d,e^	454 (47.8)^d^	155 (44.5)^d,e^	965 (48.5)	
	Female	334 (48.3)^d,f^	487 (51.2)^d^	185 (53.1)^d,f^	1006 (50.6)	
	Nonbinary	1 (0.1)^d^	9 (0.9)^d^	8 (2.2)^d^	18 (0.9)	

^a^NSC: nonsmartphone-use control latent class.

^b^ISC: ineffective–smartphone-use control latent class.

^c^ESC: effective–smartphone-use control latent class.

^d^Figures with the same exponent in each column were significantly different (*P*<.05). For example, regarding sex, 52% was significantly different from 0%; 48% was significantly different from 0%; and 52% and 48% were not significantly different.

^e^Regarding sex, 52% is significantly different from 0%.

^f^Regarding sex, 48% is significantly different from 0%.

### The Most Important Predictor Variables of the Latent Class Membership (RQ5)

**Figure 1:**
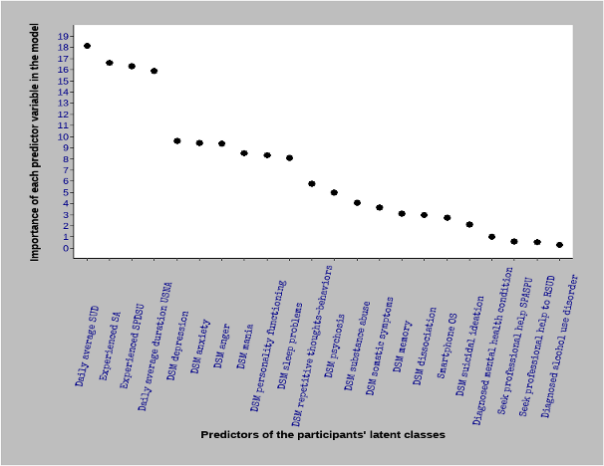
Mean decrease accuracy plot of the random forest machine learning model. It shows, in descending order of importance, the predictor variables of latent class membership. DSM: Diagnostic and Statistical Manual of Mental Disorders; OS: operating system; RSUD: reduce smartphone use duration; SA: smartphone addiction; SPASPU: social problems associated with smartphone problematic use; SPDSU: social problems owing to smartphone use; SUD: smartphone use duration; USNA: using smart phone on nonessential activities.

Figure 1 shows the RF machine learning selected model MDA plot, that is, the 22 predictor variables (in decreasing order of importance) of the 3 uncovered TMCSU latent classes.

As shown in Figure 1, of the 22 predictors included in this model, the 10 most important were daily average smartphone use, experienced smartphone *addiction*, experienced social problems owing to problematic smartphone use, daily average duration of smartphone use for nonessential activities, DSM depression, DSM anxiety, DSM anger, DSM mania, DSM personality functioning, and DSM sleep problems. The 3 least important predictor variables were alcohol use disorder diagnosis, seeking professional help to reduce smartphone use, and seeking professional help because of social problems associated with smartphone use.

As discussed, the value associated with the MDA (eg, MDA=18.11) means that if the corresponding predictor variable (ie, *daily average smartphone use duration*) were removed, the model would lose that value (ie, 18.11 points) of its total accuracy score.

The importance of the RF machine learning algorithm is that it not only enables the ranking of predictor variables but also allows for variable selection and variable multicollinearity checks. Thus, only the predictor variables with an MDA of >2 and the nonmulticollinearity variables were selected for the multinomial logistic regression model presented below.

### Associations Between the Covariates and the Three Uncovered Latent Classes (RQ6)

Table 4 summarizes the multinomial logistic regression models. In this model, the NSC latent class was set as the reference class and coded *0*, which means that the model was designed to predict the probability of an individual belonging to the ISC latent class (coded *1*) and the ESC latent class (coded *2*).

**Table 4:** Estimated β coefficients for the associations between latent classes and covariates.

Latent classes and covariates		β coefficient (SE)	OR^a^ (95% CI)	*P* value
**ISC^b^**				
	Smartphone OS^c^	−.356 (0.109)	0.7 (0.565 to 0.867)	.001^d^
	Daily average smartphone use duration	.084 (0.034)	1.088 (1.017 to 1.163)	.01^d^
	Dailly average duration using smartphone for nonessential activities	.162 (0.055)	1.175 (1.055 to 1.309)	.003^d^
	Experienced social problems owing to problematic smartphone use	.402 (0.092)	1.495 (1.247 to 1.792)	<.001^d^
	Experiencing smartphone addiction	.033 (0.012)	1.034 (1.01 to 1.059)	.006^d^
	DSM^e^ depression	.037 (0.089)	1.038 (0.872 to 1.234)	.68
	DSM anger	.156 (0.074)	1.169 (1.011 to 1.353)	.04^d^
	DSM mania	.135 (0.088)	1.144 (0.963 to 1.359)	.13
	DSM anxiety	.000 (0.089)	1 (0.839 to 1.191)	.99
	DSM somatic symptoms	−.025 (0.081)	0.975 (0.832 to 1.143)	.76
	DSM suicidal ideation	−.296 (0.119)	0.744 (0.589 to 0.939)	.01^d^
	DSM psychosis	.51 (0.243)	1.665 (1.033 to 2683)	.04^d^
	DSM sleep problems	.072 (0.054)	1.074 (0.967 to 1.193)	.18
	DSM memory	−0.019 (0.078)	0.981 (0.843 to 1.143)	.81
	DSM repetitive thoughts-behaviors	.052 (0.124)	1.053 (0.825 to 1.344)	.68
	DSM dissociation	.047 (0.108)	1.048 (0.849 to 1.295)	.66
	DSM personality functioning	−.028 (0.089)	0.972 (0.816 to 1.158)	.76
	DSM substance abuse	−.018 (0.086)	0.982 (0.829 to 1.164)	.84
**ESC^f^**				
	Smartphone OS	−.321 (0.155)	0.725 (0.536 to 0.982)	.04^d^
	Daily average smartphone use duration	.192 (0.042)	1.212 (1.117 to 1.316)	<.001^d^
	Dailly average duration using smartphone for nonessential activities	.146 (0.076)	1.158 (0.997 to 1.344)	.06
	Experienced social problems owing to problematic smartphone use	.896 (0.111)	2.45 (1.973 to 3.043)	<.001^d^
	Experiencing smartphone addiction	.061 (0.017)	1.062 (1.029 to 1.097)	<.001^d^
	DSM depression	.02 (0.122)	1.021 (0.804 to 1.295)	.87
	DSM anger	.089 (0.100)	1.093 (0.897 to 1.331)	.38
	DSM mania	.257 (0.110)	1.293 (1.042 to 1.605)	.02^d^
	DSM anxiety	.035 (0.118)	1.036 (0.822 to 1.305)	.77
	DSM somatic symptoms	.011 (0.107)	1.011 (0.819 to 1.247)	.92
	DSM suicidal ideation	−.221 (0.140)	0.802 (0.609 to 1.056	.12
	DSM psychosis	.786 (0.266)	2.195 (1.304 to 3.695)	.003^d^
	DSM sleep problems	.028 (0.074)	1.029 (0.889 to 1.19)	.71
	DSM memory	.101 (0.096)	1.107 (0.917 to 1.336)	.29
	DSM repetitive thoughts-behaviors	−.159 (0.154)	0.853 (0.631 to 1.154)	.30
	DSM dissociation	.259 (0.126)	1.296 (1.012 to 1.66)	.04^d^
	DSM personality functioning	.139 (0.112)	1.149 (0.922 to 1.432)	.22
	DSM substance abuse	−.321 (0.130)	0.725 (0.562 to 0.936)	.01^d^

^a^OR: odds ratio.

^b^ISC: ineffective–smartphone-use control latent class.

^c^OS: operating system.

^d^Significant at least at *P*<.05.

^e^DSM: Diagnostic and Statistical Manual of Mental Disorders.

^f^ESC: effective–smartphone-use control latent class.

In this table, the top part represents comparisons between NSC latent class, which is the reference class (baseline) and ISC latent class. The covariates significantly associated with the latent classes are the following: *DSM psychosis* (β=.51; odds ratio [OR] 1.665, 95% CI 1.033-2.683; *P*=.04); *experienced social problems owing to smartphone use* (β=.402; OR 1.495, 95% CI 1.247-1.792; *P*<.001); *daily average duration using smartphone for nonessential activities* (β=.162; OR 1.175, 95% CI 1.055-1.309; *P*=.003); *DSM anger* (β=.156; OR 1.169, 95% CI 1.011-1.353; *P*=.04); *daily average smartphone use* (β=.084; OR 1.088, 95% CI 1.017-1.163; *P*=.01); *experienced smartphone “addiction”* (β=.033; OR 1.034, 95% CI 1.01-1.059; *P*=.006); *smartphone Android OS* (β=−.356; OR 0.7, 95% CI 0.565-0.867; *P*=.001); and *DSM suicidal ideation* (β=−.296; OR 0.744, 95% CI 0.589-0.939; *P*=.01). A positive β coefficient indicates that an increase in the concerned covariate increases the probability of belonging to the ISC group, whereas a negative β coefficient indicates that an increase in the concerned covariate increases the probability of belonging to the NSC group. For the smartphone Android OS variable, for example, participants using the Android OS were significantly less likely (negative β coefficient) to be part of the ISC latent class. In the context of this analysis, the OR values can be interpreted as effect sizes. For example, if we take the DSM psychosis covariate, which has an OR of 1.665, for each unit increase in the participants’ DSM psychosis score, the odds of belonging to the ISC group is 67% greater after controlling for other predictors. Note that when interpreting an OR, it is important to examine how much it deviates from 1. For instance, an OR of 0.7 means that in one group, the outcome is 30% less likely. An OR of 1.66 means that in one group, the outcome is 66% more likely. However, an OR of 2 or 3.22 means that in one group, the outcome is, respectively, 2 times or 3 times more likely.

The bottom part of the table shows comparisons between the NSC and ESC latent classes. Here, the covariates significantly associated with the latent classes are *experienced social problems owing to smartphone use* (β=.896; OR 2.450, 95% CI 1.973-3.043]; *P*<.001); *DSM psychosis* (β=.786; OR 2.195, 95% CI 1.304-3.695; *P*=.003); *DSM dissociation* (β=.259; OR 1.296, 95% CI 1.012-1.66; *P*=.04); *DSM mania* (β=.257; OR 1.293, 95% CI 1.042-1.605; *P*=.02); *daily average smartphone use* (β=.192; OR 1.212, 95% CI 1.117-1.316; *P*<.001); *experienced smartphone addiction* (β=.061; OR 1.062, 95% CI 1.029-1.097; *P*<.001); *DSM substance abuse* (β=−.321; OR 0.725, 95% CI 0.562-0.936; *P*=.01); and *smartphone Android OS* (β=−.321; OR 0.725, 95% CI 0.536-0.982; *P*=.04), this means that participants using smartphones with Android OS are significantly less likely to be part of ESC latent class.

## Discussion

### Principal Findings

The results suggest that females and younger adults are more likely to show high daily total smartphone use (4-17 h) and high (>3 h) daily nonessential smartphone use. Regarding smartphone *addiction*, 13.42% (267/1989) of the participants reported experiencing it, again with females and younger adults being significantly more likely to be affected. A slightly larger percentage, 16.24% (323/1989) reported social problems attributable to smartphone use, with younger adults being statistically more likely to be in this group. The higher risk among younger females has been highlighted in several previous studies [20-23] and has been linked to higher reliance on mobile phones by young females for interpersonal, social, and safety needs [24,25]. This argues for an inclusive and sex-minded design for smartphone monitoring and control tools.

Regarding the use of TMCSU, the smartphone functionalities that limit notifications and reduce screen time were the most commonly tried and used by nearly half of the sample, followed by those that improve sleep (702/1989, 35.29%). Once tried, participants were most likely to keep using sleep-related tools (484/702, 68.9%) and those that limit notifications (436/973, 44.8%). This suggests awareness of real problems such as insomnia, distractibility, and encroachment on other aspects of life caused by disruptive and excessive engagement with smartphones and is in line with increased citizen calls for more effective regulation of *Big Tech* and up-to-date legislation to curb runaway technology growth [26]. This also reflects good acceptability of these tools, suggesting that the introduction of rigorously tested and proven alternatives in the future would likely be embraced by many smartphone users.

The need for more efficacious tools is highlighted by the finding that relatively small percentages of frequent users of tools that move apps from the home screen, delete apps, and help improve sleep actually found them effective (81/291, 27.8%; 126/574, 21.9%; and 147/702, 20.9%, respectively). Similar issues were highlighted in the experiences of parents in our sample; while more than a third targeted their underage children with tools to monitor and limit their smartphone use, and more than half relied on them frequently, only a third found them effective.

The LCA revealed intriguing results. A total of 34.74% (691/1989) of the sample mapped to a class that had a low or nonexistent probability of using any queried smartphone tools, with males, adults, and older adults being significantly more likely to belong to this group than females and younger adults. This suggests that, if proven effective, the marketing of new tools that curb excessive smartphone use should focus on these subgroups. Another 17.5% (348/1989) had a moderate to high probability of using the queried tools and tended to find them effective, with females and younger adults more likely to belong to this group than males, adults, and older adults. This suggests that females and younger adults, who in our sample were statistically more likely to experience smartphone *addiction* and to spend most of their time using their smartphone and performing nonessential smartphone activities, were also the most optimistic about the possibility of finding help on their smartphones. This is not surprising; accustomed to pursuing all activities on the web, digital natives may also gravitate toward finding help there, including for technology-mediated problems [27]. The largest latent class (965/1989, 48.52%) had a moderate probability of using the queried tools and tended to consider them ineffective, with females, younger adults, and adults being more likely to be part of this group than males and older adults.

Regarding the predictors of the extent to which participants used tools to monitor and control smartphone behaviors and whether they found them effective, no solid conclusions could be drawn. The machine learning model suggests that the most important predictors are related to smartphone use behavior, interpersonal relationships, and some psychopathological aspects (eg, daily smartphone use, smartphone *addiction*, social problems, DSM depression, DSM anxiety, DSM anger, DSM mania, DSM personality functioning, and DSM sleep), while the less important predictors are related to other psychopathological aspects (eg, alcohol use disorder diagnosis, DSM suicidal ideation, and DSM dissociation).

In addition, participants using the Android OS were more likely to not use tools to monitor or control smartphone use compared with those using iOS, perhaps suggesting inadequate marketing and outreach on the part of its maker or an inferior product or platform. Similarly, participants with high scores on the DSM suicidal ideation and substance abuse measures were more likely to not use TMCSU behavior. This may suggest a heavier reliance on smartphones among the more severely depressed or substance users, making curtailing use less appealing. Alternatively, it could suggest self-esteem– or motivation-related obstacles among participants with depression.

Other mental health conditions did not seem to discourage the use of these tools, but no clear pattern emerged as to their effectiveness. Participants with high scores on DSM mania and dissociation were statistically more likely to report effective use versus no use, possibly as a means to reduce stimulation in the former group. In contrast, those with high scores on DSM anger and total nonessential smartphone use, were more statistically likely to report ineffective use versus no use, possibly owing to the difficulty in reaching the effective threshold of tool engagement among those with heavy nonessential use or engaging appropriately with the tools among those with anger issues. In addition, some participants with high scores on *addiction*, DSM psychosis, daily average smartphone use duration, and social problems owing to use were significantly more likely to ineffectively use the TMCSU, whereas others were significantly more likely to effectively use the TMCSU.

Taken together, our data seem to portend well for the acceptability and possible effectiveness of mobile telepsychiatry help, including for conditions considered more challenging and for which digital health interventions may not have been seriously considered.

A few limitations complicate our interpretations and warrant discussion. The web-based questionnaire was based on self-reporting, which can introduce bias and compromise validity. This is true, for example, when recalling the amount of time spent, the specific tools used, and the effectiveness of the tools used. In addition, the conditions assessed—smartphone *addiction* as well as DSM-based categories—were not the product of the gold standard in-person comprehensive diagnostic evaluation and may, therefore, be unreliable. Furthermore, the sample, though large and with a broad age range and nearly equal male-female sex distribution, was exclusively US-based, potentially limiting its generalizability. The sample also included only adults aged ≥18 years, when many of the issues assessed are highly relevant to younger adolescents who are often thought to be disproportionately impacted by smartphone use and internet-related technologies. Whether our findings can be generalized to this subpopulation is unknown. In addition, the fact that our survey was exclusively on the web may also have overrepresented individuals with smartphone-related problems or those who gravitate to smartphone solutions. Furthermore, despite a study sample representative of the adult population in the United Sates across demographic variables, a selection bias related to Prolific participation or study selection by participants cannot be ruled out.

Finally, the survey was conducted during the COVID-19 pandemic, a period that witnessed heightened reliance on internet-related technologies, which likely affected participants’ engagement with and perceptions of their smartphones and smartphone tools. Nevertheless, this is the first psychological evaluation of smartphone tools that curb smartphone use, and our results suggest a potentially promising future for this digital mental health intervention.

### Conclusions

At >20 years of age, internet *addiction* has become a condition whose treatment is taking place over digital platforms. The old joke that asked users to “click here if you are addicted to the internet” is no longer funny insofar as users are increasingly “clicking” for that service as they seek on the web the tools and resources to address a problem that they are more aware of than ever before. Our study shows a relatively high acceptability of these tools and an openness to trying and using them, even if the effectiveness of the currently available tools remains inadequate. This is true for individuals trying to monitor or curtail their own use, as well as parents trying to achieve the same for their underage children. Given the limitations and despite 2 decades of research, of the psychopharmacological and psychotherapeutic offerings tested, the field and culture at large would benefit from rigorous scientific testing of these and other tools and their intelligent deployment with an eye toward those groups that seem most affected and those that seem most resistant. As it stands now, however, these tools are being developed, marketed, and widely adopted largely outside of any meaningful scientific scrutiny by the mental health field. This raises an important issue that the field must address: these built-in tools are often offered by the smartphone makers themselves and as a result come with a *built-in* conflict of interest. Digital companies rely on the amount of time users spend interacting with their products for their income. Therefore, any endorsement by smartphone makers of tools that limit smartphone use should invite some skepticism, including any public relations-type motives.

Finally, the COVID-19 pandemic has brought greater acceptability to the telepsychiatry field overall, which could mean even larger adoption of smartphone tools that are meant to enhance well-being in the future. This would constitute a clear advance if these tools can be proven effective in well-designed representative research trials and suggests that the time is ripe for such research trials to be conducted.

## Abbreviations

DSM: Diagnostic and Statistical Manual of Mental Disorders
ESC: effective–smartphone-use control
ISC: ineffective–smartphone-use control
LCA: latent class analysis
MDA: mean decrease accuracy
NSC: nonsmartphone-use control
OR: odds ratio
OS: operating system
RF: random forest
RQ: research question
TMCSU: tools to monitor and control smartphone use
